# Morphological diversity and connectivity of hippocampal interneurons

**DOI:** 10.1007/s00441-018-2882-2

**Published:** 2018-08-06

**Authors:** Sam A. Booker, Imre Vida

**Affiliations:** 1 Centre for Discovery Brain Sciences, University of Edinburgh, Edinburgh, EH8 9XD UK; 2 Simons Initiative for the Developing Brain, University of Edinburgh, Edinburgh, EH8 9XD UK; 3 Institute for Integrative Neuroanatomy, Charité - Universitätmedizin Berlin, Berlin, Germany

**Keywords:** Hippocampus, Interneuron, GABA, Morphology, Connectivity

## Abstract

The mammalian forebrain is constructed from ensembles of neurons that form local microcircuits giving rise to the exquisite cognitive tasks the mammalian brain can perform. Hippocampal neuronal circuits comprise populations of relatively homogenous excitatory neurons, principal cells and exceedingly heterogeneous inhibitory neurons, the interneurons. Interneurons release GABA from their axon terminals and are capable of controlling excitability in every cellular compartment of principal cells and interneurons alike; thus, they provide a brake on excess activity, control the timing of neuronal discharge and provide modulation of synaptic transmission. The dendritic and axonal morphology of interneurons, as well as their afferent and efferent connections within hippocampal circuits, is central to their ability to differentially control excitability, in a cell-type- and compartment-specific manner. This review aims to provide an up-to-date compendium of described hippocampal interneuron subtypes, with respect to their morphology, connectivity, neurochemistry and physiology, a full understanding of which will in time help to explain the rich diversity of neuronal function.

## Introduction

Cortical microcircuits, including the hippocampus, are critical processing units giving rise to the exquisite cognitive functions of the mammalian brain. Microcircuits comprise two major neuronal classes: excitatory principal cells (PC) and inhibitory interneurons (INs), which release the neurotransmitters glutamate and GABA, respectively. GABA exerts powerful inhibition on cells, acting as a brake for neuronal excitability through hyperpolarizing or shunting mechanisms by activating fast ionotropic GABA_A_ and slow metabotropic GABA_B_ receptors (GABA_A_Rs and GABA_B_Rs, respectively). GABA_A_Rs are primarily synaptically localized with high Cl^−^ permeability and their inhibitory potential defined by intracellular Cl^−^ concentration. Postsynaptic GABA_B_Rs in contrast are predominantly extrasynaptic and, in most neurons, preferentially activate G-protein-coupled inward-rectifying K^+^ channels postsynaptically, producing hyperpolarization. GABA_B_Rs are also found presynaptically and reduce neurotransmitter release primarily via the inhibition of voltage-gated Ca^2+^ channels. Therefore, the location of GABA release, with respect to its receptors, will determine the strength and type of inhibition provided by INs.

Indeed, while INs comprise only 10–20% of neurons within a region, they are exceedingly heterogeneous in terms of their morphology, connectivity and intrinsic and synaptic properties. The anatomical diversity of INs has already been recognized and classification into a number of types was established by early Golgi-staining studies. Their neurochemical divergence was later established by immunocytochemical investigations. However, the full examination of their heterogeneous properties has only been made possible due to advancement of intracellular electrophysiological techniques, sharp electrode and whole-cell patch-clamp recordings combined with intracellular labeling, thus allowing the combined assessment of their anatomical and physiological properties, as well as their synaptic connectivity, performed both in vitro and in vivo. Finally, the increasing use of optogenetic manipulations has allowed further identification of IN functional synaptic connectivity and their network role.

Interneurons are characterized by a dense local axon providing strong local inhibition through feedforward and feedback connections, with a small subset projecting outside their local area. Indeed, the convention of IN classification takes into account many of their functional properties critical to our understanding of their role in microcircuit function. A cardinal element in this classification is the identity of postsynaptic targets, which dissects INs into three broad classes on the basis of subcellular compartment and cell-type specificity: INs innervating the (i) perisomatic domain (“perisomatic inhibitory,” PI), (ii) dendritic regions (“dendritic inhibitory,” DI) and (iii) those preferentially targeting INs (“IN-specific” INs, IS-INs). Indeed, in hippocampal subfields with their simple laminar structure, this output connectivity is well reflected by the distribution of the axon in and near the *stratum* (*str.*) *pyramidal* versus the dendritic layers. Further classifiers include the distribution and arborization of dendrites, reflecting the afferent inputs to the neurons, their intrinsic and synaptic physiological properties and their neurochemical identity. Furthermore, in vivo studies in the last decades revealed that IN types are differentially activated in distinct behavioral states and contribute to network activity patterns.

The developmental origin of INs correlates strongly with neurochemical identity (Tricoire et al. 2011), depending on which ganglionic eminence they derive from. Furthermore, growing evidence shows that IN subtypes are highly divergent in their genetic transcript profile (Zeisel et al. 2015); however, these elements are outwith the remit of this review and have been well reviewed elsewhere (Kepecs and Fishell 2014).

INs are central to our understanding of circuit function and while they have been reviewed previously (Amaral et al. 2007; Freund and Buzsáki 1996; Klausberger 2009; Pelkey et al. 2017), these reviews have not taken into account the full complexity and connectivity of all known subtypes. This review aims to define the morphology, synaptic connectivity, neurochemical profile and electrophysiological characteristics of hippocampal INs, with respect to the local microcircuit, with a particular focus on the CA1 region. The taxonomical approach we take assumes a unique cell type if axonal and dendritic morphologies show specific laminar distributions with respect to afferent inputs to that subfield, as well as they have distinct neurochemical and physiological properties.

## Cellular and synaptic organization of the CA1 region

The hippocampus has a striking layered structure, resulting from the orderly organization of the PCs (Amaral and Witter 1989). In CA1, the somata of CA1 PCs are found in the *str. pyramidale*, giving rise to a large caliber apical dendrite extending into the *str. radiatum*, with fine oblique dendrites. This apical dendrite bifurcates in the *str. lacunosum* and forms a tuft in the *str. moleculare*. The latter two layers are often referred to together as the *str. lacunosum-moleculare* (*str. L-M*). Basal dendrites of CA1 PCs emerge from the soma and extend into the *str. oriens*. CA1 PC axons originate from the soma or a proximal dendrite (Thome et al. 2014), traverse the *str. oriens* and project along the *alveus*; a number of axon collaterals ramify within the *str. oriens* forming recurrent synapses. The main afferents arriving in CA1 are (i) the Schaffer collaterals from CA3, synapsing in the *str. radiatum* and *oriens*; (ii) temporoammonic axons from L3 of the medial entorhinal cortex (mEC), projecting to the *str. L-M*, and CA2 PC afferents, synapsing in the *str. oriens*; and (iii) CA1 recurrent axons that terminate in the *str. oriens* predominantly on INs (Takács et al. 2012). INs that predominantly receive extrinsic inputs are considered feedforward elements, while those that receive local recurrent inputs are considered feedback.

## Perisomatic inhibitory interneurons

The best described INs are perisomatic inhibitory (PI) INs, comprising basket cells (BC, axons of which target PC somata and proximal dendrites) and axo-axonic cells (AAC, targeting PC axon initial segments). PI INs, in particular BCs, have been very well studied, given their high numbers and the strong and functionally highly relevant inhibition they exert. While comprising ~ 25% of known anatomical and neurochemical IN subtypes, they make up approximately 50% of all INs, reflecting their central role in microcircuit function.

### Basket cells

#### Fast-spiking parvalbumin BCs

**Fig. 1:**
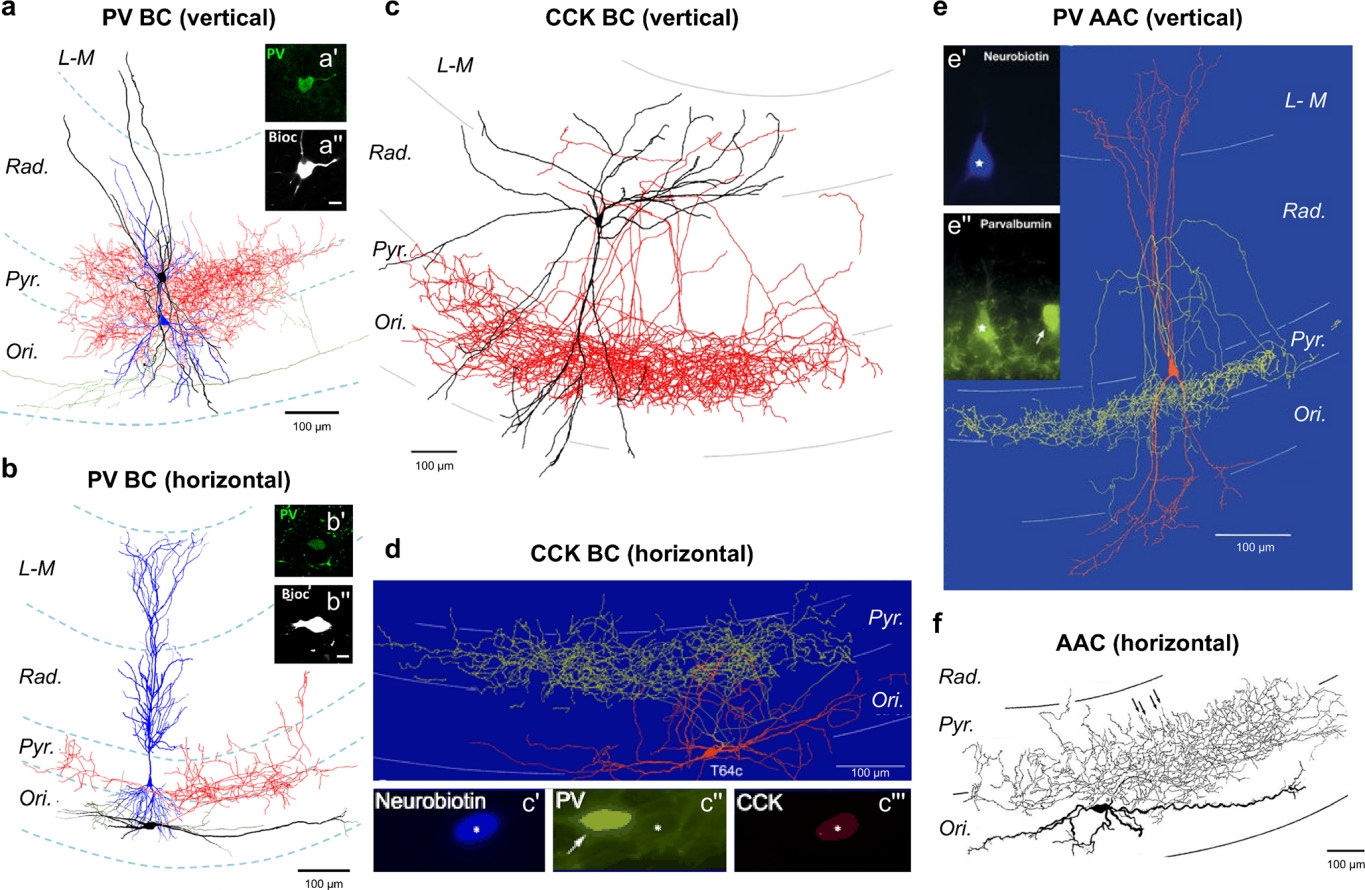
Perisomatic inhibitory INs of hippocampal subfield CA1. Example reconstructions of perisomatic inhibitory INs: PV BCs (**a**, **b**), CCK BCs (**c**, **d**) and axo-axonic cells (**e**, **f**) in the CA1 area, with either vertical (**a**, **c**, **e**) or horizontal (**b**, **d**, **f**) dendritic arbors. Soma and dendrites are shown as black (**a**, **b**, **c**, **f**) or red (**d**, **e**), while the axon is shown as red (**a**–**c**), yellow (**d**, **e**), or gray (**f**). In **a** and **b**, CA1 PCs are shown for reference (blue). Immunohistochemical labeling for PV (a′, b′, c″, and e′) and CCK (c″′) are shown as insets. All reconstructions are shown with respect to CA1 layers: *Ori*, *str. oriens*; *Pyr, str. pyramidale*; *Rad.*, *str. radiatum*; *L-M*, *str. lacunosum-moleculare*. Adapted with permission from: **a**, **b**—Booker et al. (2017); **c**—Vida et al. (1998); **d**—Klausberger et al. (2005); **e**—Klausberger et al. (2003); **f**—Ganter et al. (2004)

The most common types of BC in CA1 are those that express the calcium-binding protein parvalbumin (PV), with somata found in the *str. pyramidale* or proximal *str. oriens* and *radiatum* (Fig. 1a). PV BCs are generally fast-spiking with respect to their action potential (AP) discharge and have low membrane resistance. Dendrites of this IN type are typically vertically oriented spanning all layers of the CA1 but the extent to which they enter the *str. L-M* is unclear; recordings from the dorsal CA1 suggest minimal dendrites in that layer (Klausberger et al. 2003; Sík et al. 1995; Tukker et al. 2013), whereas recordings from the ventral CA1 indicate that up to 15% of dendrites are present (Booker et al. 2017; Gulyás et al. 1999; Lee et al. 2014). Whether this is a technical artifact or a function of the dorso-ventral axis of CA1 remains unclear. The overall dendritic length for vertically oriented PV BCs is 4347 ± 1125 μm (Gulyás et al. 1999) and they typically lack dendritic spines or are sparsely spiny but many excitatory synapses form on the dendritic shaft (3.3 synapses/μm in PV BCs versus 1.6 spine/μm in CA1 PCs) (Gulyás et al. 1999; Trommald et al. 1995). The lateral extent of a PV BC dendritic tree ranges from 377 to 875 μm along the transverse axis (Fukuda and Kosaka 2000). Overall, PV BCs receive over 10-fold more excitatory than inhibitory inputs (1055 inhibitory versus 15,238 excitatory synapses; Halasy et al. 1996), suggesting that they are highly excitable circuit elements. The axon of CA1 PV BCs arises from the soma and ramifies heavily within the local *str. pyramidale*, forming synaptic contacts with somata (~ 50%) and proximal dendrites (~ 50%) of CA1 PCs and PV BCs (Cobb et al. 1997). The lateral extent of PV BC axons is typically circular to elliptic within the *str. pyramidale*, extending 780–1740 μm from the cell body (lateral area ~ 0.3 mm^2^) forming approximately 10,000 synaptic contacts and each BC may contact ~ 1100 CA1 PCs with ~ 6 contacts per PC (Buhl et al. 1994b; Halasy et al. 1996; Sík et al. 1995). The axon of PV BC displays a preferential targeting of CA1 PCs found toward the *str. oriens* (Lee et al. 2014). PV BCs also target other PV BCs, with one in vivo labeled cell contacting 64 others (Sík et al. 1995), corresponding well to the ~ 290 PV-positive inhibitory presynaptic terminals on PV BC somata, making up 27.6% of its total GABA-positive inputs, with a strong concentration on the perisomatic domain (70% of GABAergic synapses) (Halasy et al. 1996). As in the neocortex (Ascoli et al. 2008), examples of narrow arbor (axons exclusively in the *str. pyramidale*) or wide arbor (with some axons in the *str. oriens* and proximal *radiatum*) have been observed (Pawelzik et al. 2002). In addition to the “classic” vertical PV BCs, examples of horizontal basket cells with dendrites confined to the *str. oriens* have been described (Booker et al. 2017; Lacaille et al. 1987; Pawelzik et al. 2002) (Fig. 1b). Based on their dendritic arborization, vertical PV BCs receive inputs from all major excitatory inputs to the CA1, notably: Schaffer collaterals from CA3, recurrent inputs from CA1 PCs and temporoammonic inputs from the mEC, suggesting that these INs act predominantly as feedforward signalling elements. However, paired recordings from synaptically coupled CA1 PCs and vertical PV BCs confirmed that they also receive a feedback excitatory input with a connection probability of 4.5% (Ali et al. 1998). Horizontal PV BCs receive strong input from CA1 PCs and form a distinct subpopulation of feedback BCs (Lacaille et al. 1987); the contribution of Schaffer collaterals to their input, however, is not yet established. PV BCs form robust synaptic connections onto CA1 PCs with a high, ~ 50% connection probability (Booker et al. 2013) and mutual inhibitory connections to other PV BCs with 82% likelihood (Daw et al. 2009; Fukuda and Kosaka 2000). These synaptic connections have a low 10–20% failure rate, with large amplitudes and fast kinetics (Ali et al. 1998; Booker et al. 2017). Synaptic GABA_A_Rs producing this current show low expression of γ and δ subunits, due to their reduced sensitivity to diazepam and zinc (Pawelzik et al. 1999) but contain α1, α2 and β3 subunits (Kasugai et al. 2010; Thomson et al. 2000). Due to the high-affinity calcium binding of PV itself within axon terminals, transmitter release from PV BCs is highly synchronous (Daw et al. 2009) and, in response to repeated stimuli, displays short-term depression (Booker et al. 2017). In addition, PV BCs express several key presynaptic neuromodulatory receptors, notably, muscarinic acetylcholine 2 (Hajos et al. 1997) and μ-opioid receptors (Drake and Milner 1999), with approximately 50% of PV BC terminals expressing the GABA_B_R (Booker et al. 2017), leading to reduced GABA release via inhibition of P/Q-type Ca^2+^ channels (Wilson et al. 2001). Indeed, GABA_B_R autoreceptors on PV BC axon terminals contribute to the short-term depression observed upon repetitive stimulation (Booker et al. 2017). In addition to axonal synapses, PV BCs form unique dendro-dendritic synapses, with vesicles observed in dendritic shafts forming synapses with neighboring PV BC dendrites as well as forming gap-junction electrical synapses (Fukuda and Kosaka 2000).

#### Regular-spiking cholecystokinin BCs

The other major types of CA1 BC express and release the neuropeptide cholecystokinin (CCK, Fig. 1c). CCK BCs can be subdivided based on (i) their co-expression of the neuropeptide vasoactive intestinal peptide (VIP), which have somata localized to distal *str. radiatum* (Acsády et al. 1996a, b), or (ii) those expressing VGluT3 at their axon terminals, which are more commonly found at the *str. pyramidale* and *oriens* (Somogyi et al. 2004). CCK BCs make up approximately 26% of all CCK INs (Pawelzik et al. 2002), with ~ 11% co-expressing VIP and ~ 30% expressing VGluT3 (Somogyi et al. 2004). These differences in expression and soma location do not appear to alter dendritic or axonal morphology, as CCK BCs typically have three to six radially oriented dendrites that tend to favor the vertical axis (Cope et al. 2002) and span all layers of the CA1 (Booker et al. 2016; Pawelzik et al. 2002). The dendrites are aspiny with a total length of 6338 ± 986 μm (Mátyás et al. 2004) with pronounced beading of distal dendrites noted (Pawelzik et al. 2002). The overall dendritic distribution is ~ 50% in the *str. radiatum*, 20–30% in the *str. L-M* (including *str. lacunosum*) and ~ 20% in the *str. oriens*, with a negligible proportion of dendrites in the *str. pyramidale* (Booker et al. 2016; Mátyás et al. 2004) similar to PV BCs*.* Unlike PV BCs, CCK BCs receive less excitatory and more inhibitory inputs, with an average ~ 5200 excitatory synapses and ~ 2700 inhibitory synapses and higher densities of excitatory synapse are observed on distal dendrites (Mátyás et al. 2004). This synaptic profile is distinct to of that of PV BCs (Gulyás et al. 1999) and CA1 PCs (Megıas et al. 2001), suggesting a greater inhibitory synaptic input to CCK BCs, correlating well with a high density of extrasynaptic GABA_B_Rs on their dendritic surface (Booker et al. 2016). The axon of CCK BCs ramifies heavily in the *str. pyramidale*; however, they have more collaterals in the *str. radiatum* and *oriens* than PV BCs (Pawelzik et al. 2002). Given that the cell bodies of these cells are found at high density at the s*tr. radiatum/L-M* border, the axon must traverse several hundred micrometers and segregate several times, before arborizing in the *str. pyramidale*. Long sections of their main axon collaterals are often myelinated before reaching the *str. pyramidale*, indicating increased reliability and speed of conduction (Cope et al. 2002; Pawelzik et al. 2002), a unique property among CA1 INs.

CCK BCs are typically regular spiking, with adapting trains of action potentials in response to depolarizing currents, as well as high input resistance (Booker et al. 2016). The synapses formed by CCK BCs onto CA1 PCs evoke large amplitude IPSCs (118 ± 13 pA) (Neu et al. 2007), with a high degree of temporal jitter due to the absence of a fast Ca^2+^ buffer, leading to asynchronous release properties (Daw et al. 2009; Hefft and Jonas 2005). However, axon terminals of CCK BCs highly express CB1 cannabinoid receptors (CB1Rs) and transmission is strongly modulated by retrograde endocannabinoid signaling (Wilson et al. 2001; Hefft and Jonas 2005; Neu et al. 2007). A variety of connection probabilities of CCK BC have been reported ranging from 20% (Daw et al. 2009) to > 80% (Vida et al. 1998) onto CA1 PCs and 21% to other CCK BCs (Daw et al. 2009). GABA_A_Rs present at CCK BC synapses are enriched for the α2 subunit (Nyíri et al. 2001) but also contain the α1 and β3 subunits (Kasugai et al. 2010). As for PV BCs, a small subset of CCK BCs is found in the *str. oriens* and possesses horizontal dendritic morphologies (Fig. 1d), indicating that they may in fact represent another feedback BC subtype (Maccaferri et al. 2000; Pawelzik et al. 2002). CCK BC axon terminals possess strong presynaptic neuromodulatory potential via high expression of the CB1Rs, as already indicated above but also GABA_B_Rs (Booker et al. 2017; Neu et al. 2007).

#### Axo-axonic cells

Axo-axonic cells (Fig. 1e) express PV with somatic localization very similar to that of PV BCs. The dendrites of AACs, while mostly vertically oriented and aspiny, show a pronounced dendritic tuft in the *str. L-M* (Li et al. 1992) but with shorter overall dendritic lengths (3325 μm) compared to PV BCs due to reduced proximal dendritic branching (Papp et al. 2013). AACs receive strong excitatory inputs from all major inputs to CA1 (Buhl et al. 1994a), with a similar excitatory synapse density to PV BCs (Papp et al. 2013). The axon of AACs emerges typically from the soma and forms multiple collaterals that ramify heavily in proximal *str. oriens* forming synaptic contacts with identified axon initial segments of CA1 PCs in characteristic cartridges (Somogyi et al. 1983). Each cartridge consists of 2–10 axon terminals (Li et al. 1992), with a total of 23–93 symmetric inhibitory synapses formed per axon initial segment (AIS) (Kosaka 1980), suggesting that each CA1 PC AIS could be contacted by 15–20 AACs; however, the number is more likely closer to 6 (Buhl et al. 1994b) with a single AAC contacting up to 1300 CA1 PCs (Buhl et al. 1994a). In good agreement, synaptic connectivity of AACs is very high when measured from paired recordings (Buhl et al. 1994b; Cobb et al. 1995). AACs produce large amplitude IPSCs through activation of α2 containing GABA_A_Rs (Nusser et al. 1996). The functional effect of this synaptic contact is contentious, as it has been proposed that dynamic Cl^−^ concentrations in the axon initial segment may lead to either excitatory or inhibitory synaptic responses (Dugladze et al. 2012; Glickfeld et al. 2009; Szabadics et al. 2006). As for BCs, examples of horizontal AACs have been identified (Fig. 1f), with strong unitary connections from CA1 PCs, confirming that both forward and feedback subtypes exist (Ganter et al. 2004).

## Dendritic inhibitory INs

INs that target the dendritic domain of PCs and other INs are the most diverse population in the CA1 region (Klausberger 2009). With unique dendritic and axonal distributions, neurochemical profiles and electrophysiology, they are able to inhibit CA1 PCs in a pathway-specific manner, either inhibiting a single layer, or with axons spanning multiple layers and occasionally leaving CA1 entirely to inhibit long-range targets (Melzer et al. 2012).

### Bistratified cells

**Fig. 2:**
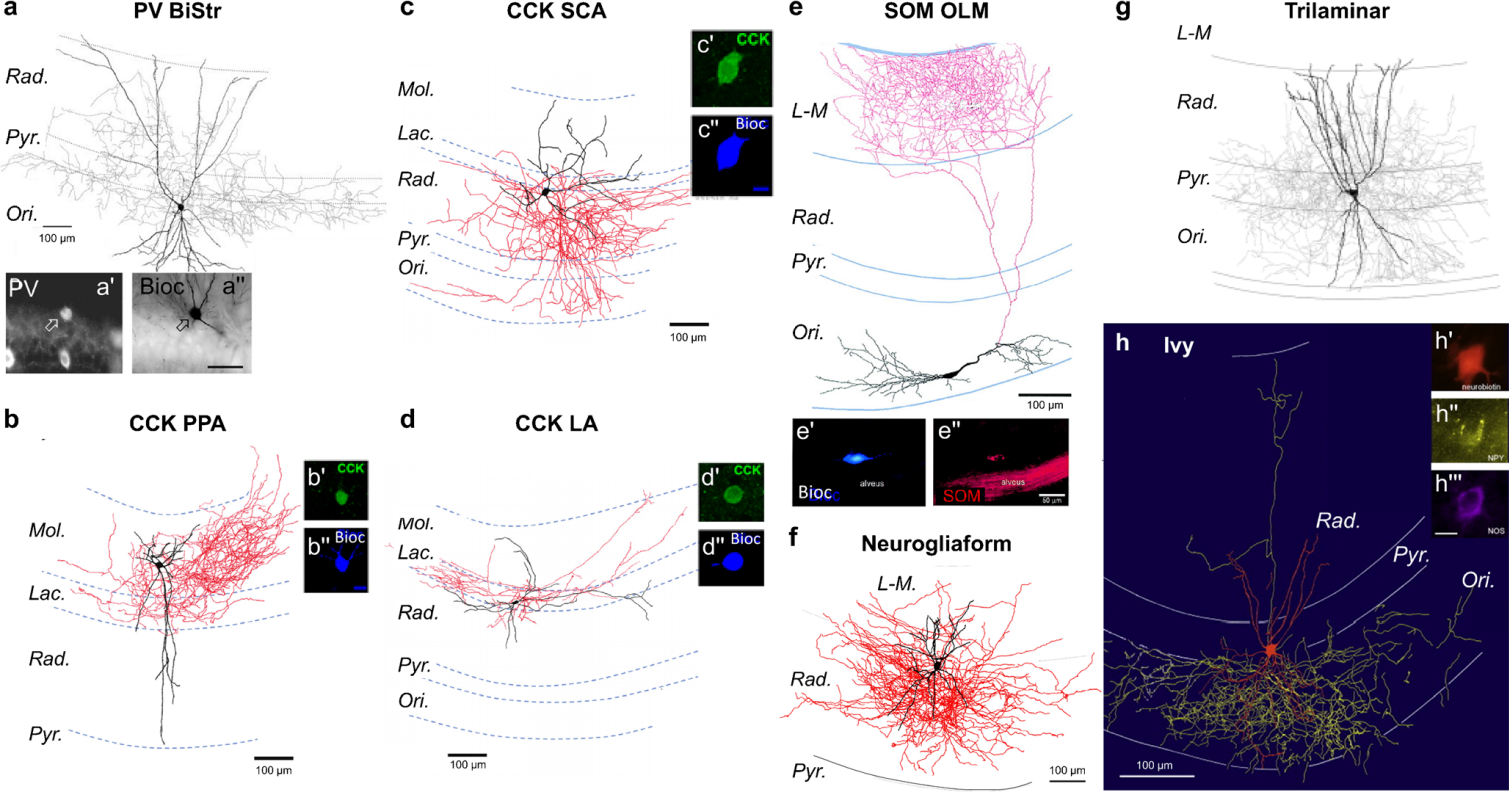
DI INs of hippocampal subfield CA1. Examples of CA1 DI INs. Soma and dendrites are shown as black (**a**–**g**) or red (**h**), while the axon is shown as gray (**a**, **g**), red (**b**–**f**), or yellow (**h**). Immunohistochemical labeling for PV (a′), CCK (b′, c′, d′), SOM (e″) and NPY (h″) and nNOS (h″′) is shown as insets. All reconstructions are shown with respect to CA1 layers: *Ori*, *str. oriens*; *Pyr*, *str. pyramidale*; *Rad.*, *str. radiatum*; *L-M*, *str. acunosum-moleculare*. Adapted with permission from: **a**, **g**—Pawelzik et al. (2002); **b**–**d**—Booker et al. (2016); **e**—Martina et al. (2000); **f**—Vida et al. (1998); **h**—Fuentealba et al. (2008)

DI cells constitute between 16 and 25% of all PV INs (Bezaire and Soltész 2013; Pawelzik et al. 2002), also expressing somatostatin (SOM) (Baude et al. 2006; Klausberger et al. 2004) and are referred to as bistratified cells (BiStr, Fig. 2a) (Buhl et al. 1994b). With somata located close to the *str. pyramidale*, vertical BiStr cells have vertically oriented dendrites extending from the *str. oriens* to *radiatum*, with few dendrites in the *str. L-M* (Halasy et al. 1996; Pawelzik et al. 2002) and as such receive a strong input from Schaffer collaterals (Buhl et al. 1994b) and also CA1 PCs (14.3% connection probability) (Ali et al. 1998). BiStr cells possess an axon that emerges from the soma or proximal dendrite and ramifies heavily within the *str. oriens* and *radiatum* proximal to the *str. pyramidale*, with a wide lateral extent: 1860 μm septo-temporal and 2090 μm medio-lateral. The total axon length is 78,800 μm, forming approximately ~ 16,600 synaptic contacts with up to 2500 local CA1 PCs (Sík et al. 1995), leading to a high ~ 33.3% connectivity with CA1 PCs (Pawelzik et al. 2002). Unitary connections in one identified BiStr were mediated by six spatially distributed synapses on shafts of medium and small caliber oblique apical and basal dendrites of the PC (Buhl et al. 1994b). These synapses produce typically fast IPSCs, which are smaller and slower when recorded at the soma than for PV BCs (Booker et al. 2013), likely due to dendritic filtering.

Furthermore, a second population of BiStr cells has horizontal morphologies, referred to as the *str. oriens*-bistratified (O-BiStr) cells and is distinct from the vertical type above. Horizontal BiStr cells express both SOM and PV (Booker et al. 2018) and have a dendritic arbor similar to other SOM-INs (see below) and are strong feedback inhibitory circuit elements. Their axon ramifies heavily in the *str. oriens* and *radiatum* but not in the *str. L-M*, forming synaptic contacts with apical dendrites and small secondary dendrites, with up to 10 synaptic contacts between SOM BiStr-CA1 PC pairs (Maccaferri et al. 2000). SOM BiStr cells produce robust IPSCs in CA1 PCs and provide sustained inhibition in response to repetitive trains of stimuli, due to an absence of short-term depression (Maccaferri et al. 2000).

### SOM *oriens*/*lacunosum-moleculare* cells

INs in the *str. oriens* that express the neuropeptide SOM are regarded as the prototypical feedback IN (Fig. 2e; Katona et al. 1999; Lacaille et al. 1987). SOM *oriens*/*lacunosum-moleculare* (OLM) cells are one of the most studied CA1 INs as they represent an archetypal dendritic inhibitory IN and constitute the majority of horizontal fusiform somata at the *str. oriens/alveus* border. Their horizontally oriented dendrites run parallel to the alveus, often forming dendritic tufts in the alveus (Lacaille et al. 1987; Maccaferri et al. 2000; Sík et al. 1995). In CA1, the dendrites of OLM cells rarely, if ever, cross the *str. pyramidale* and are densely covered with long, thin dendritic spines (Maccaferri et al. 2000), which receive synaptic contacts (Martina et al. 2000). The axon of OLM cells usually originates from a primary dendrite and extends into the *str. L-M* where it ramifies heavily (> 90% of axon terminals), forming synapses with dendrites and spines of CA1 PCs (Maccaferri et al. 2000; Martina et al. 2000). In some OLM cells, the axon gives rise to local collaterals in the *str. oriens*, constituting ~ 7% of all terminals and may also extend into the subiculum (1.5% of terminals; Sík et al. 1995). Despite this, the lateral spread of OLM cell axons is relatively small (840 × 500 μm). The total length of the axon is over 63,000 μm, indicating a high density of OLM cell axon in their projection zone (Martina et al. 2000; Sík et al. 1995). The excitatory synaptic inputs to OLM cells arise almost entirely (> 75%) from local CA1 PCs (Blasco-Ibáñez and Freund 1995), which produce robust EPSPs in OLM cells (Maccaferri and McBain 1995; Ouardouz and Lacaille 1995; Topolnik et al. 2005), with a connection probability of 33% (Ali and Thomson 1998). This synapse shows strong facilitation in response to repetitive stimuli, a feature defined by both pre- and postsynaptic mechanisms (McBain et al. 1994). With high expression of mGluR1α on their dendrites, OLM cell activity is dependent on the spillover of synaptic glutamate to induce synaptic plasticity and Ca^2+^ influx (Baude et al. 1993; Topolnik et al. 2005, 2006). Surprisingly, OLM cells receive a mostly homogenous local inhibitory input from calretinin (CR)/VIP IN-specific INs (IS-INs type 3; see below), which have ~ 34% connectivity onto identified SOM OLM cells; but multiple CR/VIP IS-INs are required to inhibit OLM cell action potential discharge (Tyan et al. 2014). Interestingly, the strength of inhibition onto SOM OLM cells increases throughout development, due to increased membrane insertion of the α5 GABA_A_ subunit (Salesse et al. 2011). As stated above, CA1 PC distal dendritic shafts and spines are the main targets of OLM cells and as such, stimulation of the alveus (driving OLM cell activity) has been shown to strongly hyperpolarize CA1 distal dendrites (Samulack et al. 1993; Yanovsky et al. 1997). Despite the long electrotonic distance of OLM synapses from CA1 PC somata, a couple of in vitro studies have shown unitary connections and were reported to have low connection probabilities of 6.7%; however, this value is likely an underestimate due to the slicing procedure (Maccaferri et al. 2000; Minneci et al. 2007). Inhibitory responses produced by OLM cells in CA1 PCs are typically small with slow kinetics. This is unexpected given that up to seven synapses are involved in producing the response (Maccaferri et al. 2000) but could be explained by extensive dendritic and spine neck filtering of GABA_A_ currents (Jayant et al. 2017). In addition to expressing SOM, ~ 50% OLM cells also express PV at low levels (Booker et al. 2018; Maccaferri et al. 2000), which may underlie the strong paired pulse depression at their output observed in response to repetitive stimuli (Minneci et al. 2007). SOM INs can produce heterosynaptic spillover and activation of GABA_B_Rs (Nichol et al. 2018; Urban-Ciecko et al. 2015). Despite few examples, a subpopulation of SOM INs is present in the *str. radiatum*, projecting to the *str. L-M* with vertically oriented dendrites, suggesting a potential feedforward subtype (Oliva et al. 2000).

### CCK DI cells

CCK DI cells are the most morphologically diverse of all DI cells, with at least seven identified subtypes in the CA1 alone. Despite such diversity, many of the subtypes have not been fully analyzed. All CCK DI cells are regular spiking in nature, with spike train adaptation observed consistently.

#### Schaffer collateral-associated INs

CCK Schaffer collateral-associated (SCA) cells have somata located across all layers of the CA1 (Fig. 2c) but with notable enrichment at the *str. radiatum/L-M* border (Dudok et al. 2015; Vida et al. 1998). The dendrites of SCA cells are multipolar and span all layers of the CA1, with the greatest density of dendrites in the *str. radiatum* but with dendrites in the *str. L-M* and to a lesser degree in the *str. oriens* (Booker et al. 2016; Cope et al. 2002; Hájos and Mody 1997; Vida et al. 1998). The axon of SCA cells typically emerges from a proximal dendrite and run parallel to the *str. radiatum/L-M* border along the transverse axis giving rise to multiple collaterals that ramify heavily in the *str. radiatum* (55%) and *str. oriens* (35%) (Booker et al. 2016). The axon has a lateral spread of ~ 1100 μm consisting of ~ 6000 boutons within an acute slice (400 μm), which represents approximately 50% of the total axon labeled in vivo (Vida et al. 1998). SCA cells form 4–6 putative synaptic contacts with CA1 PCs, on small caliber dendrites and occasionally dendritic spines. The majority of boutons (70%) are on spiny, putative PC dendrites, suggesting that each SCA cell could contact up to 1400–2100 CA1 PCs (Vida et al. 1998); as such, the connection probability of SCA cells is high at 65%. As well as targeting CA1 PCs, SCA cells also form inhibitory synapses with other SCAs with a connection probability of 8.3% (Ali 2007). Furthermore, occasional electrical coupling via gap-junctions has been observed between SCA cells at an incidence of 2.1% (Ali 2007). Indeed, SCA INs have been shown to inhibit other INs, located within the *str. pyramidale* with up to 30% of synapses formed on aspiny putative IN dendrites; thus, SCA cells could contact 400–700 other INs within the CA1 (Vida et al. 1998). SCA cells evoke small IPSCs with amplitudes of ~ 20 pA, which are strongly depressing in response to repetitive stimuli and have a 40% failure rate (Booker et al. 2017). In addition to CCK, SCA cells express calbindin in their cytosol (Cope et al. 2002) and CB1Rs on their axon terminals giving rise to low probability release (Ali and Todorova 2010; Ali 2007). Furthermore, they express presynaptic GABA_B_ autoreceptors that also strongly inhibit their output (> 95%), thus efficiently controlling the SCA network (Booker et al. 2017).

#### Apical dendrite-associated INs

Apical dendrite-associated (ADA) cells bear a similarity to SCA cells with respect to somatodendritic organization but unlike SCA cells, their axon is restricted to the *str. radiatum* (Hájos and Mody 1997) where it preferentially forms synapses with large caliber apical dendrites (Klausberger et al. 2005). The synapses formed by ADA cell axon terminals are significantly larger than those of other CA1 INs. They highly express CB1Rs as well as VGluT3 (Klausberger et al. 2005), consistent with their CCK neurochemistry.

#### *Str. oriens-str. oriens* INs

These neurons are minimally identified; however, cells expressing CCK with similar somatic morphology may comprise 5% of all CCK neurons (Pawelzik et al. 2002). *Str. oriens-str. oriens* (SO-SO) cells have a horizontal multipolar dendritic tree and axonal arbor entirely confined within the *str. oriens*. The single SO-SO cell reconstructed had ~ 1800 axon terminals, which contacted the basal dendrites of CA1 PCs and had reciprocal connectivity with a local CA1 PC. This cell had very high levels of spontaneous excitatory input (51 Hz) suggesting a high synapse density, plausibly from local PCs (Pawelzik et al. 2002). Given the high level of connectivity and location of dendrites and axon, SO-SO cells are positioned to act as strong feedback elements.

#### CCK trilaminar INs

With an inverted pyramidal-shaped soma located within the *str. radiatum*, the singular described CCK trilaminar cell possessed a high density (50%) of axon terminals in the *str. radiatum*, as well as projecting into the *str. oriens* (~ 25%) and *pyramidale* (~ 25%). Despite being regular spiking and containing CCK, nothing further is known of this cell type (Pawelzik et al. 2002).

#### Quadrilaminar INs

Quadrilaminar cells have ovoid somata located at the *str. radiatum/L-M* border, with multipolar dendrites spanning all layers. Their axon is concentrated in the *str. radiatum* and *str. L-M* but had significant ramifications in the *str. pyramidale* and *oriens*, too. One of the two identified quadrilaminar cells synaptically coupled to a CA1 PC with two putative synaptic contacts, which produced a small but minimally depressing IPSC (Pawelzik et al. 2002). Blockade of GABA_A_Rs revealed a small GABA_B_R conductance, consistent with a dense axon capable of producing focal volume transmission (Scanziani 2000).

#### Perforant path-associated INs

The somata of perforant path-associated (PPA) cells are typically found in the deep *str. radiatum* with radially oriented aspiny dendrites that span from the *str*. *radiatum* to the hippocampal fissure; no dendrites traverse the *str. pyramidale* (Fig. 2b; Booker et al. 2016; Klausberger et al. 2005; Pawelzik et al. 2002; Vida et al. 1998). Over 70% of PPA cell axon is found in the *str. L-M*, with several collaterals passing into the dentate gyrus, the subiculum and occasionally into the *str. oriens* of CA1. The axon was found to form synaptic contacts with dendritic shafts and form up to ~ 8000 synaptic contacts (Vida et al. 1998). Direct unitary connectivity of PPA INs onto CA1 PCs has not been observed in paired recordings (Vida et al. 1998), likely due to the small amplitude of IPSPs and the large electrotonic distance between synapse location and PC somata. However, PPA cells appear to couple to SCA cells electrically via gap-junctions, with a coupling probability of 4–6% (Iball and Ali 2011). Nothing is known of the excitatory input to PPA cells but they receive a rich inhibitory input from the *str. radiatum*, *str. L-M* and potentially dentate gyrus (Khazipov et al. 1995) and the exact cellular source of this inhibition remains unknown.

#### *Lacunosum*-associated INs

With somata located within the *str. lacunosum*, lacunosum-associated (LA) cells have horizontal, aspiny dendrites running along the transverse axis of the CA1, confined to distal *str. radiatum* (50%) and *str. L-M* (Booker et al. 2016; Fig. 2d)*.* Unlike PPA neurons, examples of which have been described with a similar dendritic tree (Pawelzik et al. 2002), the LA IN axon extends horizontally along the same transverse axis, with > 90% of the axon found within the *str. lacunosum* or the distal region of the *str. radiatum*. As well as CCK, LA cells express presynaptic CB1Rs, consistent with other CCK INs. LA cells have a very large voltage “sag” in response to hyperpolarizing currents, indicating a large contribution of *I*_h_ to their membrane excitability (Booker et al. 2016). The high degree of overlap of the LA cell axon at the location of the main CA1 PC dendritic bifurcation may indicate a role in inhibiting that compartment specifically.

### Neurogliaform cells

Neurogliaform cells (NGFCs) expressing neuronal nitric oxide synthases (nNOS) are found typically in the distal *str. radiatum* and *str. L-M* (Fig. 2f), although examples have been observed closer to the *str. pyramidale*. Their very small, round somata give rise to several aspiny multipolar dendrites, which each repeatedly bifurcate close to the cell body (~ 20–30 μm), not extending more than ~ 100 μm radially from the soma in a dense plexus (Price et al. 2005; Vida et al. 1998). As this structure resembles glial cells, they were named accordingly (Vida et al. 1998). The axon of NGFCs is extremely compact and dense with a lateral spread of 500–700 μm and up to 1200 μm septally, containing approximately 13,000 axon terminals targeting dendritic shafts (58% of terminals) and spines (15% of terminals) of putative CA1 PCs (Fuentealba et al. 2010; Vida et al. 1998). However, a high proportion of axon terminals do not appear to couple to a postsynaptic element (Oláh et al. 2009; Vida et al. 1998). In addition to the dense local axon, NGFCs also send axon collaterals across the hippocampal fissure to the dentate gyrus (Price et al. 2005; Vida et al. 1998), thus allowing coordinated inhibition between the two hippocampal subfields.

NGFCs receive a strong excitatory input from entorhinal inputs within the *str. L-M*, which due to their compact electrotonic nature is capable of efficiently discharging them. A major source of inhibition to NGFCs is from other NGFCs (Price et al. 2005), with a connection probability of ~ 75%. The GABA_A_R IPSCs produced at this unitary connection are reliable, with amplitudes of 21 ± 20 pA and a very slow decay in the range of several tens of milliseconds (Price et al. 2005). As well as contacting other NGFCs, these INs strongly inhibit the distal dendrites of CA1 PCs producing similarly slow IPSCs (Price et al. 2008; Vida et al. 1998), due to the presence of α5 and δ subunits in postsynaptic receptors (Karayannis et al. 2010). Due to the very dense axon of this IN type, they are also capable of unitary volume transmission activating extrasynaptic GABA_B_Rs, in both CA1 PCs and local INs (Booker et al. 2013; Oláh et al. 2009; Price et al. 2008). In addition to chemical connections, NGFCs form a tight electrically coupled network, with 34–85% connection probability through gap-junctions (Price et al. 2005; Zsiros and Maccaferri 2005).

### Ivy cells

A more recently described IN subtype are the Ivy cells, expressing nNOS and neuropeptide-Y (NPY), named on the basis of the vine-like appearance of their axonal arbor (Fig. 2h) (Fuentealba et al. 2008). The soma of Ivy cells are located proximal to the *str. pyramidale* with up to six radial dendrites extending into the *str. radiatum* and minimally into the *str. oriens* and *L-M* (Fuentealba et al. 2008; Somogyi et al. 2012; Tricoire et al. 2010)*.* These INs have 2000–3500 μm of axon found within the *str. oriens*, *pyramidale*, *radiatum* and minimally in *L-M*, primarily forming synaptic contacts with oblique branches and basal dendrites of CA1 PCs (81% of boutons), with 13% of synapses formed on spines and 6% directly on main apical dendrites (Fuentealba et al. 2008; Somogyi et al. 2012). Ivy cells form reliable unitary connections with CA1 PCs (60% connection probability) and CA1 PCs also synapse onto them with ~ 80% probability. Unitary EPSPs and IPSPs recorded in these neurons were typically broader than in BiStr cells (Fuentealba et al. 2008). The interaction between Ivy cells and other INs is not yet known.

### Trilaminar cells

With somata located in the *str. oriens*, trilaminar cells have either horizontal (Sík et al. 1995) or radial (Ali et al. 1998) dendrites (Fig. 2g). In the horizontal orientation, they resemble SOM INs. The axon of the *str. oriens* trilaminar cells resembles those described above (section “CCK trilaminar INs”), having a long (56,000 μm, 15,767 boutons) and dense axon within the *str. oriens* (12.8%), *pyramidale* (16.7%) and *radiatum* (68.4%); it should be noted that axons in the *str. pyramidale* formed a significant proportion of synaptic contacts in this layer (Sík et al. 1995), with up to 19% of synapses contacting PC somata (Ferraguti et al. 2005). Little is known of the connectivity of trilaminar neurons but they receive a strong excitatory Schaffer collateral input as well as strong inhibition (Sík et al. 1994, 1995). In addition to the dense local axon, trilaminar cells may also produce long-range retrohippocampal connections to CA3 and dentate gyrus (DG) (see also “INs with local and long-range projecting axons” section), ramifying in the dendritic fields of principal cells (Sík et al. 1994). It remains, however, unknown if this is a characteristic of all trilaminar cells or of only a subset.

## IN-specific INs

**Fig. 3:**
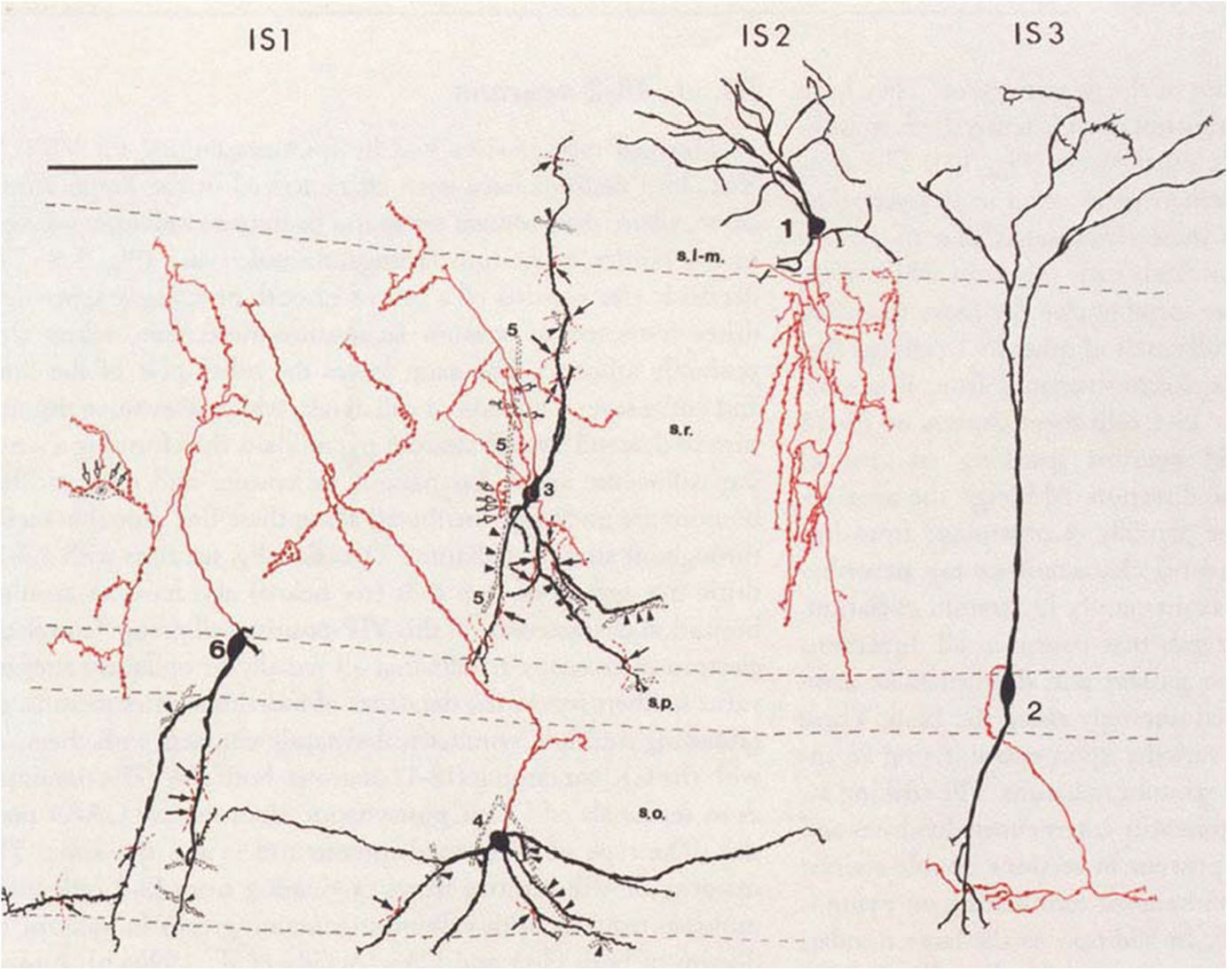
IN-specific INs of hippocampal subfield CA1: distribution of CR immunoreactive IS-INs with respect to somatodendritic axis (black) and axonal arborisation (red). Reproduced with permission from Freund and Buzsáki (1996)

A large set of CR and/or VIP expressing IS-INs form a unique niche among INs, as they selectively inhibit other INs. As described above, most INs, while preferentially innervating PCs, can also inhibit other INs. However, IS-INs preferentially target the dendritic domains of INs and can thereby produce robust disinhibition in the local microcircuit (Tyan et al. 2014). IS-INs can be divided into three subtypes, based on their neurochemistry and morphology (Fig. 3).

### IS-IN type I

Type I IS-INs express CR but not VIP and the somata are mostly found in the *str. oriens*, *pyramidale* and *radiatum*, where they often form clusters of five to six neurons. They have small round somata and aspiny, vertical dendrites. Their axon collaterals are primarily localized within the *str. radiatum*, where they form contacts with CB- and CR-immunoreactive dendrites (Acsády et al. 1996a, b). While no study has assessed their functional connectivity, their dendritic and axonal organization suggests that they provide feedforward inhibition to the *str. radiatum* INs. Immunocytochemical studies show that they from mutual inhibitory synapse with other IS-INs and target CB-positive dendrite-inhibiting interneurons but avoid PV-expressing BCs and axo-axonic cells (Acsády et al. 1996a, b; Gulyás et al. 1996). Additionally, they form mutual inhibitory and close dendro-dendritic contacts, with dendrites bundled and coupled by electrical synapses, plausibly synchronizing their output within their local cell cluster (Acsády et al. 1996a, b).

### IS-IN type II

Expressing both CR and VIP, type II IS-INs typically have somata in the *str. L-M*, with unipolar, aspiny dendrites that branch heavily in the *str. L-M* (Gulyás et al. 1996). Similar to IS-IN type I, the axon projects primarily to the *str. radiatum*, where they form contacts with CR-immunoreactive dendrites (Acsády et al. 1996a, b). Thus, they contribute heavily to the mutual inhibitory CR IN network but most likely do not receive inhibition from this network. In addition to CR-positive targets, IS-IN type I innervates CB- and CCK-immunopositive INs. Their dendritic and axonal organization suggests that they provide feedforward disinhibition, driven primarily by perforant path afferents, to the *str. radiatum* INs. No quantitative functional connectivity data are available for this cell type to date.

### IS-IN type III

The last subtype of IS-IN, so called type III, express CR, VIP (Tyan et al. 2014) and nNOS (Tricoire et al. 2010). They have somata in the *str. pyramidale* and *radiatum*, with two to five vertical, bipolar dendrites spanning all layers and form a tuft in the *str. L-M* (Acsády et al. 1996a, b; Chamberland et al. 2010). The axons of type III IS-INs typically emerge from a dendrite in the *str. radiatum* and ramify exclusively in the *str. oriens* forming synaptic connections selectively with SOM-INs in that layer with a connection probability of 36% (Chamberland et al. 2010). The IPSCs produced by type III IS-INs are typically small and show no short-term plasticity at low frequencies but facilitate at 100 Hz stimulation (Tyan et al. 2014).

## INs with local and long-range projecting axons

Within CA1, a population of INs possesses both local and long-range projecting axons, targeting other hippocampal subfields, retrohippocampal regions and the septum. These INs are referred to as projection INs and are believed to synchronize oscillatory activity across brain regions, allowing coordinated neuronal firing. Despite these perceived roles, very little is known of the cellular function or the local and long-range functional connectivity of projection INs.

### RADI cells

RADI projection INs express CB and COUP-TFII and have somata in the *str. L-M*, with short dendrites that remain in that layer (Fuentealba et al. 2010). The axon of RADI cells densely innervates the *str. radiatum*, forming synaptic contacts with the dendrites of CA1 PCs and other INs but minimally ramifies in the *str. L-M*. RADI cells send an axon collateral across the hippocampal fissure to the *str. granulosum* of the DG, forming BC-like synapses with the cell bodies of dentate granule cells (Fuentealba et al. 2010).

### *Oriens*/retrohippocampal projection

*Str. oriens* projection INs are better defined, with several studies examining them. Their bipolar, horizontal dendrites are confined to the *str. oriens*, indistinguishable from other horizontal *str. oriens/alveus* IN types. *Oriens* retrohippocampal projection INs possess a dense axon in the *str. radiatum* and *oriens* of CA1, where they predominately contact CA1 PC dendrites (Jinno et al. 2007). The large and heavily myelinated projection axon of these INs ramifies in either subiculum (Jinno et al. 2007) or the mEC where it preferentially forms synapses onto local INs (Melzer et al. 2012). *Oriens*/retrohippocampal INs are immunoreactive for CB, as well as SOM (Oláh et al. 2009).

### *Radiatum*/retrohippocampal projection

With somata located at the *str. radiatum/L-M* border region and with dendrites located in these layers, *radiatum*/retrohippocampal INs have an axon that may form local synapses in the *str. L-M* but sends a thick, myelinated axon to the subiculum, presubiculum, retrosplenial cortex and the indusium griseum, where the axon preferentially forms synapses with IN dendrites (Jinno and Kosaka 2003). Little else is known about these INs.

### Backprojecting I (SOM)

A subpopulation of SOM, NADPH-diaphorase and NOS expressing INs possesses sparsely spiny, horizontally oriented dendrites at the *str. oriens/alveus* border with a very long axon (> 100 mm) that ramifies heavily in the *str. oriens* and *radiatum* of CA1 (59.4% of axon), as well as 40.6% of axon traversing to the *str. radiatum* of CA3 and the hilus of DG (Goldin et al. 2007; Sík et al. 1994, 1995). The major axon targets of SOM backprojecting cells appear to be putative PC dendrites (Sík et al. 1995). Given the bistratified distribution of their axon in CA1, it remains unclear whether BiStr or trilaminar cells identified in ex vivo slice preparation may be overlapping with this cell type.

### Backprojecting II (CRH)

INs expressing CRH form an overlapping population with other neurochemical subtypes, co-expressing PV, SOM, CR, CB, or CCK. CRH INs have somata found within the *str. pyramidale* and proximal *str. oriens* (Yan et al. 1998). CRH-INs possess dendrites similar to CA1 PCs as they have large ovoid somata that give rise to basal dendrites in the *str. oriens* and apical and oblique dendrites in the *str. radiatum*, with distal dendrites invading the *str. L-M*; however, they are aspiny and express both GABA and GAD-67 (Hooper and Maguire 2016). Their axons backproject to CA3 (Hooper and Maguire 2016) where they contact the perisomatic region of CA3 PCs (Yan et al. 1998) providing strong monosynaptic inhibition (Hooper and Maguire 2016). This novel cell type appears to typify the BC subtype, albeit in a backprojection manner.

### Double projecting INs

With somata located in the *str. oriens*, double projecting INs were initially identified following retrograde labeling of the septum with horseradish peroxidase. Double projection INs express both CB and/or SOM (Gulyás et al. 2003; Tóth and Freund 1992), with 70% of them being immunoreactive for both (Gulyás et al. 2003). They have horizontal dendrites in the *str. oriens*; however, some multipolar and vertical examples have been observed. Double projecting neurons have a retrohippocampal axon that extends into CA3 and DG and a long-range septal axon. The major divergence of double projecting cells is with respect to their target cells. While some double projecting neurons contact spiny principal cell dendrites (Jinno et al. 2007), others have been shown to exclusively contact IN dendrites (Gulyás et al. 2003). Therefore, it remains ambiguous to what extent these cells are one class or multiple subtypes.

**Fig. 4:**
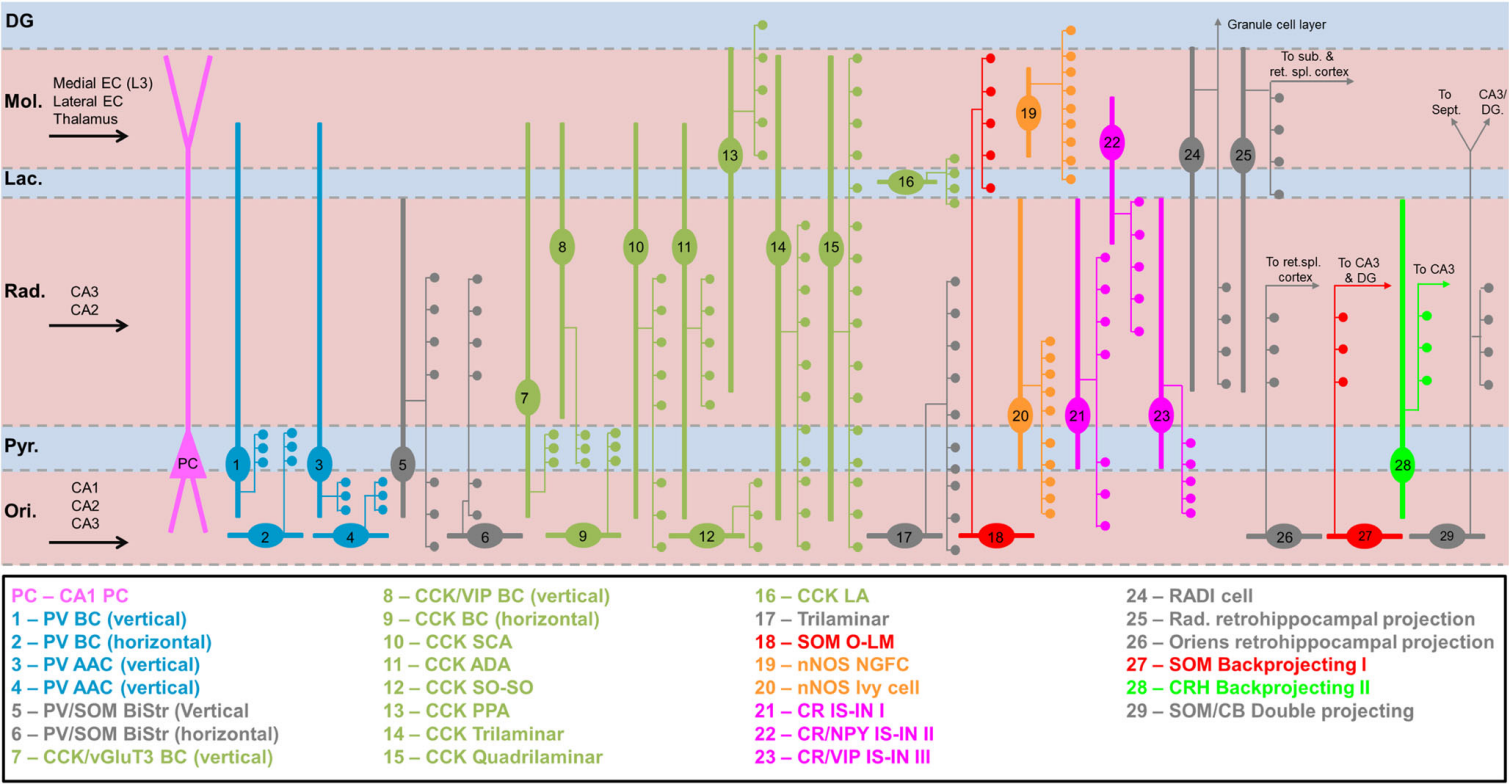
Summary of all described IN subtypes in hippocampal subfield CA. Schematic overview of known morphological and neurochemical IN subtypes in CA1. Somatodendritic domains (thick lines), axonal locations (thin lines) and major terminal fields (circles) are shown with respect to regional layers (thin dashed lines). Afferent inputs are indicated with black arrows. Layers: *Ori, str. oriens*; *Pyr*, *str. pyramidale*; *Rad.*, *str. radiatum*; *Lac.*, *str. lacunosum*; *Mol.*, *str. moleculare*; DG, dentate gyrus

In summary, CA1 INs are highly diverse based on morphology, neurochemistry and connectivity. As such, we define, based on current information, at least 29 known subtypes with distinct properties (Fig. 4), which is a substantial increase from previous estimates.

## Hippocampal subfield-specific differences

While an exceeding diversity of IN subtypes exists within CA1, many of these subtypes show a strong convergence between hippocampal subfields. However, regional differences in morphology, neurochemistry and connectivity exist in the other hippocampal subfields due to divergent laminar structure, particularly in the DG. We describe below the known differences in IN properties between the regions as well as regional specific subtypes.

### Hippocampal subfield CA3

With a very similar structure to the CA1, the CA3 subfield comprises PCs as the main principal cells, albeit with substantial morphological difference from CA1 PCs. The layering is similar, with the exception of the mossy fiber projection originating from DG granule cells forming a tight bundle in the *str. lucidum*, located between the *str. pyramidale* and *radiatum.* Furthermore, given that the CA3 PCs form their associational-commissural collateral projections within the CA3 itself, the main synaptic input is in to the *str. radiatum* and *str. oriens*. Accordingly, INs found in these layers can act as both feedforward and feedback elements. The CA3 recapitulates many of the subtypes of IN present in CA1 (Tukker et al. 2007); however, several key differences exist in IN morphology and several unique subtypes are present within the CA3.

#### SOM OLM cells

**Fig. 5:**
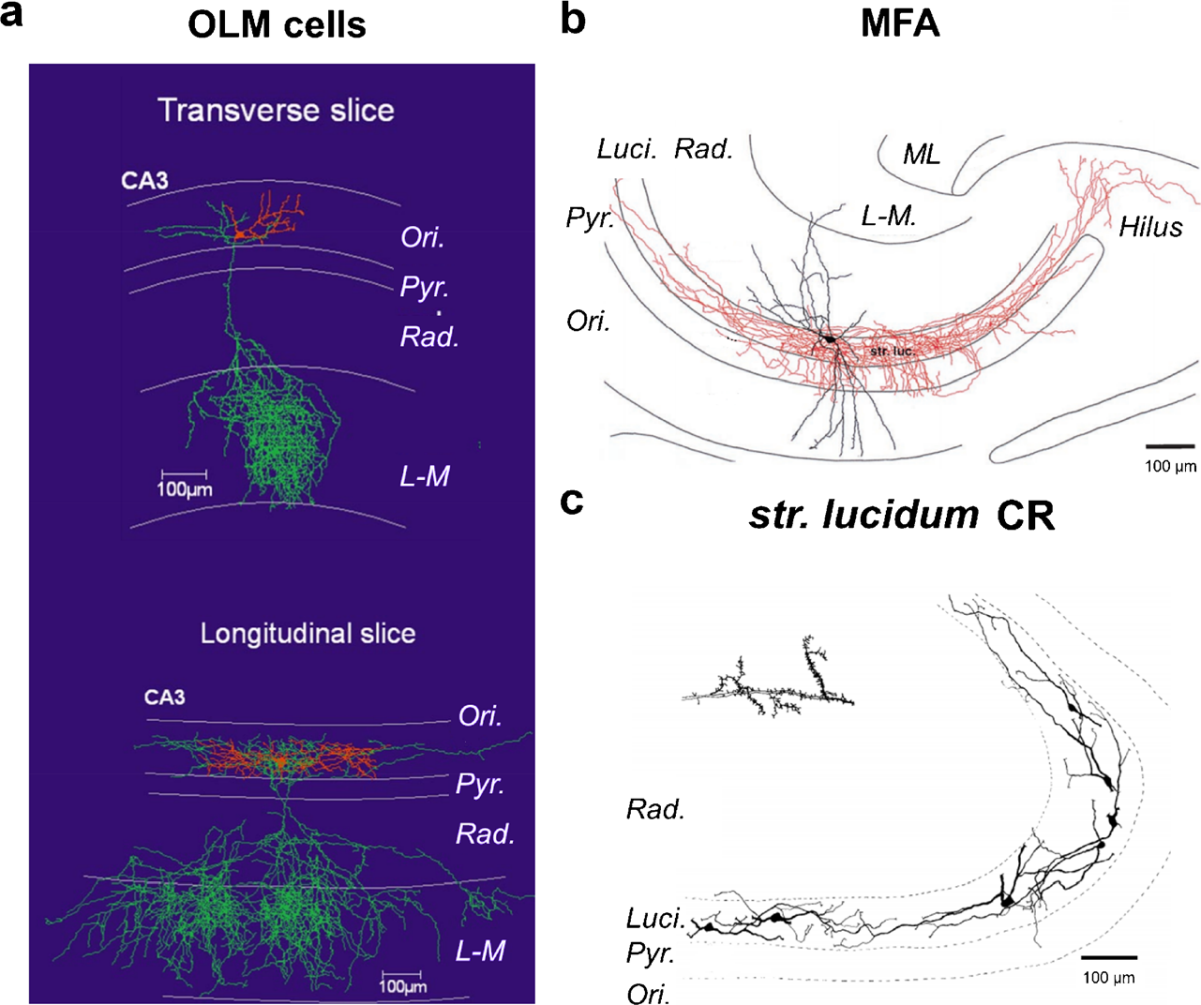
Divergent IN subtypes in hippocampal subfield CA3: reconstructions of CA3 INs showing divergence from CA1. Soma and dendrites are shown as red (**a**) or black (**b**, **c**), with the axon shown in green (**a**) or red (**b**). CR indicates calretinin immunoreactivity. Reconstructions are shown with respect to CA3 laminae: *Ori., str. oriens*; *Pyr.*, *str. pyramidale*; *Luci.*, *str. lucidum*; *Rad.*, *str. radiatum*; *L-M*, *str. lacunosum-moleculare*; ML, molecular layer. Adapted with permission from: **a**—Tort et al. (2007); **b**—Vida and Frotscher (2000); **c**—Gulyás et al. (1992)

A major region-specific difference between the CA1 and CA3 is with respect to the properties of SOM INs. The axon of CA3 OLM cells is not distributed uniformly, in that the transverse axis (223 μm spread) has less spread than the dorsal-ventral axis (1200–1300 μm spread; Fig. 5a) (Tort et al. 2007). Furthermore, there is an increased number of SOM-immunoreactive somata in the *str. radiatum* of CA3 (Sloviter and Nilaver 1987), perhaps relating to an increased number of SOM OLM cells in that layer relative to the CA1 (Oliva et al. 2000). Interestingly, it may suggest that the localization of SOM INs is strongly dependent on local recurrent inputs, as provided by CA3 PCs to the *str. radiatum*.

#### CCK DI INs

While CCK DI IN subtypes in the CA3 are mostly consistent with those of CA1, some variation exists. This is particularly noted with respect to their axonal plexus, with several examples of DI cells possessing a dendritic tree confined to CA3 but with extensive axonal arborisation in the molecular layer of DG (Lasztóczi et al. 2011).

#### Mossy fiber-associated INs

Mossy fiber-associated (MFA) INs have somata found exclusively within the *str. lucidum* and have vertically or radially oriented beaded dendrites (with occasional spines) that were localized to the *str. oriens* and *radiatum* (Fig. 5b) (Spruston et al. 1997; Vida and Frotscher 2000). The axon of MFA cells covers 50–100% of the lateral extent of mossy fiber tract and is found primarily within the *str. lucidum* (69.8%) or proximal *str. pyramidal* and *oriens* (25.6%) but minimally in the *str. radiatum* (4.6%). The total axon length of MFA cells is 24,500 μm, which make symmetric contacts every ~ 4 μm, suggesting that up to 7000 synapses form on proximal apical dendrites (85% of axon terminals) and somata (15% of axon terminals) of CA3 PCs and occasionally with other INs (Vida and Frotscher 2000). Synapses produced by MFA cells onto CA3 PCs showed large amplitude GABA_A_R-mediated IPSCs, with a mean amplitude of 284 pA, which were minimally depressing during repetitive stimuli (Vida and Frotscher 2000). Little is known of the excitatory input to MFA cells; however, they receive very strong inhibition via recruitment of inhibition in response to mossy fiber stimulation (Banke and McBain 2006). Given the high level of inhibition provided by unitary connections from MFA and their probable input from dentate granule cells, they likely contribute to the high inhibitory drive observed in response to mossy fiber activation (Banke and McBain 2006; Mori et al. 2004). MFA INs express CCK and thus represent a CA3-specific form of CCK DIs (Losonczy et al. 2004). They also express high levels of CB1R in their synaptic terminals maintaining a very low initial release probability (Losonczy et al. 2004).

#### Spiny *str. lucidum* cells

Spiny *str. lucidum* INs express CR (Fig. 5c), with somata and densely spiny dendrites running parallel to the *str. pyramidale*, rarely leaving the *str. lucidum* (Gulyás et al. 1992; Spruston et al. 1997). Their spines each receive 4–6 asymmetric contacts within the *str. lucidum* from *en passant* synapses formed by the mossy fibers, while dendritic shafts were also contacted by *en passant* synapses and also occasionally by large mossy fiber boutons. Unusually, this IN subtype appears to possess very few, if any, symmetrical inhibitory synapses on their dendrites. The axons of spiny *str. lucidum* INs make symmetric contacts with dendrites in the *str. radiatum* and *str. oriens*; at this time, it is unclear whether these *str. lucidum* spiny CR INs contact dendrites of CA3 PCs or other INs. This IN type was characterized in immunocytochemical and in vitro slice studies and may overlap with or correspond to the CR/SOM hippocampo-septal cell type described below.

#### CR/SOM hippocampo-septal cells

While the *str. oriens* septal double projection INs are present in CA3, with similar properties to those in CA1, a distinct population of projection INs with such dual projection exists (Gulyás et al. 2003). These unique double projection INs have somata localized to the *str. lucidum* and express both SOM and CR and have spiny dendrites (Gulyás et al. 2003). Interestingly, in addition to possessing a septal projection, these CA3 hippocampo-septal cells target exclusively INs in all layers of CA3 and the DG hilus (Gulyás et al. 2003). While potentially representing a long-range IS-IN subtype, it remains unclear whether CR/SOM hippocampo-septal cells, identified by retrograde tracing (Gulyás et al. 2003), form an overlapping group with the spiny CR *str. lucidum* INs described above.

### Hippocampal subfield CA2

The very narrow CA2 subfield (~ 200–300 μm transverse extent) has noticeable differences between CA1 and CA3 given that they receive few mossy fiber inputs but they also form reliable recurrent connections (Mercer et al. 2012b) that preferentially target the *str. oriens* of CA1 (Shinohara et al. 2012). With respect to local INs, one major difference between CA2 and CA1 is the number of neurochemically identified IN subtypes as compared to the total IN population, with fewer PV INs and more SOM and Reelin INs. Despite this, there is a greater density of PV INs in *str. pyramidale* and *oriens* in CA2 compared to CA1 (Botcher et al. 2014).

#### Divergent PV BC morphology and inhibition

Given the narrow transverse extent of CA2, it is unsurprising that the dendritic and axonal arbors of PV BCs extend beyond the subfield borders, innervating CA1 and/or CA3 PCs, while also receiving inputs from both regions (Mercer et al. 2007). Of interest is the presence of two morphologically defined PV BC subtypes, with narrow or wide profiles. Narrow arbor baskets have a narrow vertical dendritic arbor (312 ± 121 μm, transverse axis) compared to their wide arbor counterparts (570 ± 111 μm) and also have high *I*_h_. The axon of narrow arbor PV BCs has a transverse extent of 616 ± 130 μm, similar to that of their CA1 counterparts (681 ± 150 μm), while wide arbor baskets have a much greater lateral extent of 937 ± 133 μm, with significant innervation of both CA1 and CA3 subfields. Similar to CA1 BCs, CA2 PV BCs form mutual inhibitory synapses, with 11% connection probability, which appears to be similar between wide and narrow arbor subtypes (Mercer et al. 2007, 2012b). The dendrites of CA2 (and also CA3) PV BCs show almost a 6-fold higher dendritic length in the *str. L-M* (3025 ± 1014 μm) compared to those of CA1 PV BCs (Tukker et al. 2013). Furthermore, both narrow and wide arbor CA2 BCs receive a homogenous synaptic input from local PCs with a high connection probability of 20% (Ali et al. 1998). However, the two types diverge in their physiological properties in that PC inputs onto narrow arbor BCs are strongly depressing, while inputs onto wide arbor BCs are strongly facilitating (Mercer et al. 2012b). This is reminiscent of the target specificity of short-term plasticity observed at synapses of CA1 PCs-BCs versus PC-OLM cells (Lacaille et al. 1987), which may suggest some overlap with this population, although the presence of SOM in them has yet to be tested. Finally, CA2 PV INs provide very strong inhibition onto CA2 PCs and thus gate CA3 inputs, with unique plasticity properties dependent on delta-opioid receptors (Piskorowski and Chevaleyre 2013).

#### *Str. pyramidale*-*str. radiatum* cells

At present, one subtype of IN, unique to CA2, has been described: *str. pyramidale*-*str. radiatum* (SP-SR) cells. Somata are located in the *str. pyramidale* with several vertical dendrites extending into the *str. oriens*/alveus and *str. radiatum* but not the *str. L-M.* The dendritic span is 438 ± 144 μm vertically and 496 ± 184 μm laterally, with a total length of 4200 μm. They possess an axon that ramifies nearly exclusively in the *str. radiatum* with a total length of 15,000 μm, contacting the apical dendrites of CA2 PCs (Mercer et al. 2012a). SP-SR cells form one to two putative synaptic contacts with CA2 PCs with a 9% connection probability, producing IPSPs similar to those of BiStr cells (Mercer et al. 2012a). Meanwhile, SP-SR cells are regularly contacted by CA2 PCs, with a connection probability of 17%, producing facilitating EPSPs. Given these properties, SP-SR cells show overlap with BiStr cells and CCK ADA cells but lack expression of either PV or CCK. Further study is clearly needed to fully characterize this subtype of IN.

### The dentate gyrus

The dentate gyrus is the most divergent of the hippocampal subfields with respect to neuronal population, layering and synaptic connectivity. The monopolar dendritic morphology of dentate principal cells, the granule cells (DGC), is found in the molecular layers (ML) and their axons extend from the soma into the hilus. The DG has specific afferents that arise from layer 2 of the medial and lateral EC (outer and middle ML, respectively) and the commissural/associative inputs from hilar mossy cells (inner ML). The output of DGCs is the mossy fiber, which also produces hilar collaterals preferentially targeting INs and the mossy fiber bouton synapse with CA3 PCs (Henze et al. 2000). The diversity of IN subtypes in the DG is markedly different from that of the CA1–3 regions, albeit with similarities in their principles. Previous morphological studies and reviews have identified at least four classes of DG IN, which have distinct morphology (Amaral et al. 2007; Hosp et al. 2014; Houser 2007). Here we will highlight key differences from CA1–CA3 INs, primarily describing nine distinct and well-established types identified on the basis of anatomical and neurochemical criteria.

#### PV BCs

**Fig. 6:**
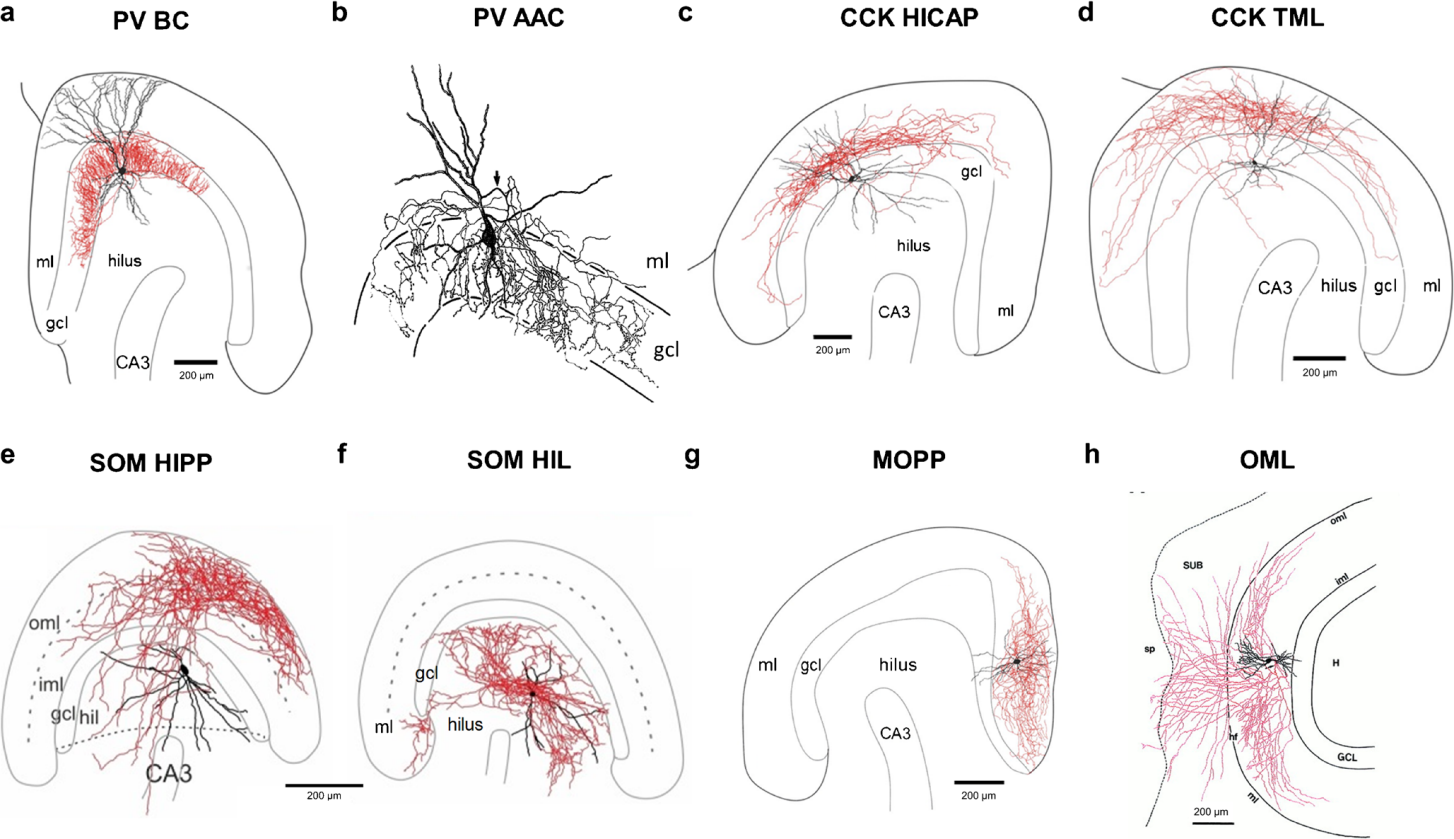
IN subtypes of the dentate gyrus: examples of morphological subtypes of identified IN in the DG of mice and rats. All panels except for **b** show soma and dendrites in black and axon in red. **b** Dendrites as thick black lines and axon as thin lines. Adapted from: **a**, **c**, **d**, **g**—Hosp et al. (2014); **b**—Soriano and Frotscher (1989); **e**, **f**—Yuan et al. (2017); **h**—Ceranik et al. (1997)

Overall, the morphology of DG BCs is broadly similar to that of CA1 **(**Fig. 6a). One major distinction is that somatic morphologies have been well characterized as falling within five major classes: pyramidal, fusiform, inverted fusiform, molecular layer and horizontal (Ribak and Seress 1983), all, however, with similar dendritic morphologies. PV BC dendrites span all layers from the oML to the hilus, receiving excitatory inputs from all major afferents and can, thus, provide both feedforward and feedback inhibition to DGCs and other INs. The morphology of vertical DG PV BCs is well described, with a total dendritic length of 3878–5423 μm (Doischer et al. 2008; Hosp et al. 2014; Nörenberg et al. 2010), of which ~ 25% is accounted for by basal dendrites in the hilus and ~ 75% for the apical dendrites in the ML (Nörenberg et al. 2010), indicating preferential inputs from ML compared to mossy fibers (Blasco-Ibanez et al. 2000). DG PV BC dendrites possess nonuniform properties, with proximal dendrites having 10-fold lower resistance than distal dendrites, which will also strengthen distal synaptic inputs (Nörenberg et al. 2010). The axon of DG PV BCs originates from the soma and forms a very dense plexus in the granule cell layer (GCL), making baskets of collaterals around the somata of DGCs. The total length of the axon is in the range 23,000–33,000 μm and can cover > 50% of the DG transverse axis over both blades (Doischer et al. 2008; Nörenberg et al. 2010). These properties show age dependence, with both axon and dendrite developing over the first 3 postnatal weeks (Doischer et al. 2008). PV BCs connect onto DGCs with a very high rate of > 50% connection probability (Doischer et al. 2008) and strong mutual inhibitory synapses with 10–15% connection probability (Doischer et al. 2008; Savanthrapadian et al. 2014), as well as being connected electrically (Doischer et al. 2008).

In addition to PV BCs, a smaller subset of DG PV PIs corresponds to axo-axonic cells (Fig. 6b). Similar to PV BCs, their properties are thought to largely correspond to axo-axonic cells to other areas but very little data are available from these INs.

#### CCK BCs

CCK BCs in the DG are poorly defined, less so than in either CA1 or CA3, with no study unequivocally identifying them. One study has suggested that the main CCK axon field only partially overlaps with that of PV BCs in the GCL, with dense labeling found in the inner molecular layer (iML) (Hefft and Jonas 2005). Given that the iML is the target of hilar commissural/associational path (HICAP) cells (see below), it can be argued that DG CCK BCs and HICAP cells constitute one subtype. Thus, the existence of CCK BCs in the DG needs to be clarified in future studies.

#### CCK hilar commissural/associational path

As mentioned above, the highest density of CCK axon terminals in the DG is found in the iML (Hefft and Jonas 2005) and belongs to HICAP cells **(**Fig. 6c) (Halasy and Somogyi 1993; Han et al. 1993; Hosp et al. 2014; Savanthrapadian et al. 2014; Soriano and Frotscher 1993), which have pyramidal-shaped somata found at the hilus/GCL border, vertically oriented dendrites present in the hilus and the ML. HICAP dendrites have a distal ML tuft, while the hilar dendrites branch less profusely and appear sparsely spiny (Sík et al. 1997). Their axon emerges from the soma, has a total length of 9700 ± 2000 μm (Mott et al. 1997) and ramifies predominantly within the iML (75.5% of axon) and the GCL (22.6% of axon) (Hosp et al. 2014; Sík et al. 1997). The lateral extent of the HICAP axon is up to 1500 μm, inhibiting over half the transverse extent of the DG (Han et al. 1993; Sík et al. 1997) and the dorso-ventral axonal extent covers almost the entire DG (2580 μm) (Sík et al. 1997). The axons of HICAP cells predominantly target large caliber apical dendritic shafts and somata of DGCs, forming symmetrical inhibitory synapses. However, some axons contact dendritic spines, where they appear to form both symmetric (60% of contacts) and asymmetric synapses (40%) from the same axon collateral (Halasy and Somogyi 1993). Even if these divergent axon terminations and postsynaptic structure indicating an underlying difference in neurotransmission from HICAP cells remain to be characterized, some hilar INs can provide an excitatory drive to mossy cells (Scharfman et al. 1990). Inhibitory synapses of HICAP cells form robust connections with asynchronous release properties onto DGCs (Harney and Jones 2002; Hefft and Jonas 2005). Furthermore, HICAP cells form mutual inhibitory synapses with 25.9% probability and strongly innervate PV BCs with 12.8% connection probability. In contrast, HICAP cells do synapse onto hilar INs (Savanthrapadian et al. 2014). The synapses of HICAP cells onto other HICAP and BCs were weak, compared to those onto DGCs, suggesting only loose synchronization formed by mutually inhibitory networks.

#### CCK total molecular layer cells

The other major CCK IN subtype in the DG is the total molecular layer (TML) cells, which bear resemblance to CCK DI subtypes in the CA1 (Fig. 6d). With somata found typically at the GCL/hilar border, TML cells have pyramidal-shaped somata giving rise to hilar basal dendrites and apical dendrites in the ML, with a total length of 2100 ± 400 μm (Mott et al. 1997; Soriano and Frotscher 1993). The hilar dendrites of TML cells receive rich innervation from mossy fiber *en passant* synapses, while the densely spiny ML dendrites receive the same inputs as DGCs (Sík et al. 1997), inferring a role in both feedforward and feedback inhibition (Soriano and Frotscher 1993). TML cell axons emerge from the soma or a proximal dendrite and run parallel to the GCL for several hundred microns, giving rise to multiple collaterals that innervate the whole extent of the ML over both inferior and superior blades of the DG (Mott et al. 1997; Soriano and Frotscher 1993). The total length of TML cell axons is 5100 ± 1200 μm (Mott et al. 1997), with a lateral extent of up to 1200 μm (Soriano and Frotscher 1993). Electron microscopy indicated that TML cells predominantly contact spiny dendrites of DGCs, mainly on their dendritic shafts (73% of axon terminals) but occasionally adjacent to or on dendritic spines (Soriano and Frotscher 1993) and TML cells also synapse onto hilar INs (Yu et al. 2015). There is no published physiological account of synaptic connectivity between TML cells and DGCs; however, given the high number of connections formed and the length of the axon, they should be assumed to be numerous. Connections between TML cells and hilar INs with dendrites in ML have been recently reported (Yu et al. 2015). These recordings showed that TML synapses possess high failure rates and produce facilitating IPSCs, which are sensitive to CB1R activation (Yu et al. 2015).

#### SOM hilar perforant path cells

With somata in the polymorphic layer of the hilus, SOM-immunoreactive hilar perforant path (HIPP) cells have spiny horizontal, bipolar dendrites, which do not cross the GCL (Fig. 6e) (Han et al. 1993; Sík et al. 1997). HIPP cells have a dendritic length of 2500 to 3200 μm (Mott et al. 1997; Zhang et al. 2009) and their arbor spans 800–900 μm in the transverse plane, with minimal spread along the septal axis (400 μm; Sík et al. 1997). The large irregular spines of HIPP cells contain functional synapses (Halasy and Somogyi 1993). The axon of HIPP cells emerges mostly from a proximal dendrite and it bifurcates and traverses the GCL, branching heavily in the oML where 75% of the axon forms synapses with the distal dendrites of DGCs (Halasy and Somogyi 1993; Han et al. 1993; Sík et al. 1997; Yuan et al. 2017). Surprisingly, a small percentage of the axon (1.7%) remains within the hilus and forms contacts with local INs (Savanthrapadian et al. 2014; Yuan et al. 2017). The lateral extent of the oML axon was 3100 μm, with a total length of 22,500 to 26,800 μm (Sík et al. 1997; Yuan et al. 2017). The estimated number of axon terminals was 76,800, indicating that HIPP cells may form synapses onto 20% of DGC dendrites within their axonal fields. HIPP cells also form a significant number of contacts onto PV dendrites in the oML, with an estimated 1355 synapses, with each PV dendrite receiving one to seven synapses (Sík et al. 1997). Consistent with the anatomical estimates, synaptic coupling from HIPP cells to DGCs has been found to have a 17.8% connectivity rate and these synapses show slow kinetics and low failure rates (Zhang et al. 2009). Furthermore, HIPP cells form contacts with other HIPP cells with a connection probability of 5.2%. These synapses, due to their hilar location, have rapid kinetics (Savanthrapadian et al. 2014). HIPP cells also form functional synapses with PV BCs with a probability of 12.8%, with synaptic responses generally having slow kinetics due to the location of these synapses in the mML (Savanthrapadian et al. 2014).

#### SOM hilar-associated IN cells

With dendritic morphology similar to HIPP cells, the SOM-immunoreactive hilar-associated IN (HIL) cells have very similar dendritic lengths (2547 ± 428 μm) located entirely within the hilus (Fig. 6f), receiving a high proportion of their synaptic inputs from mossy cells. The defining property of HIL cells is that their axon is mainly localized to the hilus (91%), with minimal invasion of the GCL (2.1%). The axon has a length of 20,700 ± 2500 μm (Yuan et al. 2017), bearing similarity to a previously described hilar IN (Han et al. 1993). These INs provide strong perisomatic inhibition onto hilar INs, including PV INs (Yuan et al. 2017). Furthermore, HIL cells also possess a long-range axon projection to the medial septum, where they provide strong inhibition to septal glutamatergic neurons (Yuan et al. 2017), in opposition to other hippocampal INs projecting to the septum (Gulyás et al. 2003). Given that the medial septum provides powerful temporal control of hippocampal activity (Sainsbury and Bland 1981), HIL cells may serve to synchronize activity between hippocampal subfields via the septum.

#### Molecular layer perforant path cells

Molecular layer perforant path (MOPP) cells have somata located in the outer half of the ML, with several smooth or sparsely spiny dendrites that run horizontally in the ML (Fig. 6g) but do not cross the hippocampal fissure (Han et al. 1993). They possess an axon that also remains in the outer two thirds of the ML and forms symmetric synapses with putative DGC and IN dendrites (Halasy and Somogyi 1993). Given the absence of dendrites in either the hilus or iML, MOPP cells produce pure feedforward inhibition in DGCs, both newborn and mature (Li et al. 2013). No further information is available on their connection properties.

#### Outer molecular layer cells

Outer molecular layer (OML) somata are found in the oML with a few, very short, aspiny, radial dendrites, which occasionally extend into the hilus but never cross the hippocampal fissure (Fig. 6h). The axon of OML cells ramifies heavily in the oML with an estimated number of 667 ± 434 synapses formed in that region but also crosses the hippocampal fissure into the subiculum forming 947 ± 657 synapses. The span of the axon is 1000–1300 μm in the transverse plane. The subicular axon targets the molecular (95% of axons) or the PC layers (5% of axon terminals). OML cells form symmetrical output synapses onto spiny dendritic shafts and spines (Ceranik et al. 1997). Nothing is known of the input connectivity of OML cells but based on their dendritic field, they likely act as feedforward signaling elements, capable of synchronizing dendritic excitability across multiple regions.

#### Neurogliaform cells

DG NGFCs are, in many respects, similar to those of CA1 and CA3. Unlike descriptions in CA1, however, DG NGFCs have been identified in both the ML (Armstrong et al. 2011) and hilus (Markwardt et al. 2011). NGFCs found in the ML have typical short dendrites with less than 100-μm span. The axon of DG NGFCs is similar to that of CA1 counterparts, branching profusely proximal to the somata. Axon collaterals often extend across the fissure and arborize in the *str. L-M* of the CA1 or the molecular layer of the subiculum (65% of cells). The axonal spread covered 37.7% (1100 μm) of the transverse extent of the DG, where synapses were formed with DGCs. The connection probability of this synapse was high, at 54% and NGFCs produced large, slow IPSCs composed of both GABA_A_R- and GABA_B_R-mediated currents. The primary afferent to DG NGFCs is the perforant path, consistent with the feedforward role of these INs (Armstrong et al. 2011). Unlike in CA1, some NGFCs have been observed in and around the GCL, where they formed synaptic contacts onto mature and immature DGCs, coordinating their activity (Markwardt et al. 2011).

**Fig. 7:**
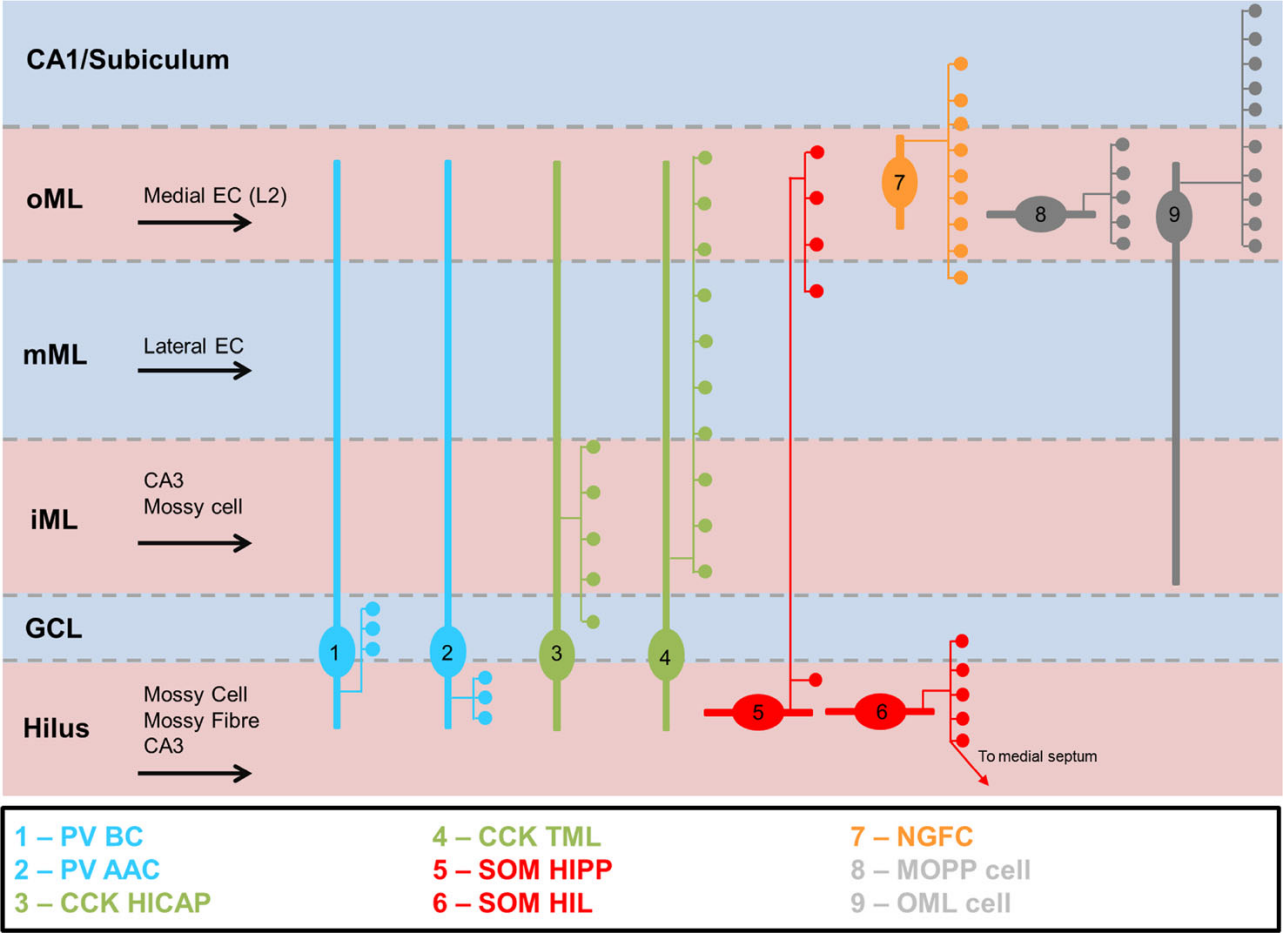
Summary of identified IN subtypes in the DG. The known morphological and neurochemical subtypes of IN in the DG are depicted, with typical localization of their somata, laminar distribution of their dendrites (thick lines) and axon distribution (thin lines) with major terminal fields (circles) with respect to DG layers (dashed lines): GCL, granule cell layer; iML, inner molecular layer; mML, medial molecular layer; oML, outer molecular layer; hippocampal fissure (thick dashed line)

In summary, DG INs, while being classified according to the classic taxonomical approach, conform to a limited number of IN subtypes (Fig. 7). Whether a higher diversity exists with potential further subtypes remains yet to be determined.

## Functional implications of diverse morphology and connectivity

While this review has largely audited the known morphology and connectivity of hippocampal INs, these properties are central to understanding how INs and PCs interact in hippocampal microcircuits to generate complex network behavior and information processing. This final section will highlight the functional consequences of their divergent morphology and connectivity in the context of the function of the hippocampus and cortical microcircuits.

### Dynamic balance of excitation and inhibition

INs are key to maintaining the balance of excitation and inhibition, which is essential for the proper function of cortical circuits (Van Vreeswijk and Sompolinsky 1996) and the brain as a whole (Yizhar et al. 2011). At the level of single cells, this balance is highly dynamic and shows a fine spatial and temporal pattern, arising from the distribution of excitatory and inhibitory synapses over the membrane surface and the activity of presynaptic neurons, most importantly diverse local INs (Li et al. 2013). Indeed, as we describe, IN types provide compartment-selective inhibition. Accordingly, the differential activation of BC, AACs, or the different DI types can produce selective inhibition, respectively, at the soma, axon initial segment, or dendrites of target PCs and INs. Fast GABA_A_R-mediated inhibition produced by these distinct types can thereby locally interact with excitatory inputs, modulate synaptic plasticity and control the integration and amplification of postsynaptic potentials or the initiation and backpropagation of action potentials (Andreasen and Lambert 1999; Davies et al. 1991; Dugladze et al. 2012; Sambandan et al. 2010; Vida et al. 2006). Thus, INs inhibit the entire membrane surface of their postsynaptic targets and collectively control its electrical activity.

### Spillover of GABA and heterosynaptic inhibition

Beyond synaptic inhibition occurring focally at anatomical contacts, INs can also release GABA into the extracellular space (“spillover”; Isaacson et al. 1993) and produce volume transmission to activate extrasynaptic GABA_B_Rs (Kulik et al. 2003) and high-affinity GABA_A_Rs (Farrant and Nusser 2005). In this respect, key features of most INs are a dense local axon and the lack of glial ensheathing of GABAergic synapses. While unitary volume transmission of GABA has been best described at NGFC synapses (Armstrong et al. 2011; Price et al. 2005, 2008), strong evidence suggests that other identified IN subtypes contribute to this form of inhibition (Booker et al. 2013; Nichol et al. 2018; Scanziani 2000; Urban-Ciecko et al. 2015). Extrasynaptic GABA_B_Rs are found on the entire somatodendritic domains (Degro et al. 2015), suggesting that sustained repetitive activation of any IN type could produce their activation (Degro et al. 2015; Scanziani 2000). While this form of transmission lacks the high spatial selectivity of “point-to-point” synaptic transmission and is limited to the laminar volume of the axonal, it has the ability to synchronize large numbers of cells to the activity of that IN subtype.

The co-alignment of IN axonal fields and specific excitatory afferents within different layers further implies specific interactions, which can take place not only postsynaptically (see above) but also presynaptically, between glutamatergic and GABAergic axon terminals. Indeed, heterosynaptic GABAergic inhibition of excitatory projections is present in the hippocampus as well as the cortex: GABA released in the *str. lucidum* of CA3, plausibly through “spillover” from MFA IN terminals, can heterosynaptically inhibit the transmission at the mossy fiber-CA3 PC synapse via presynaptic GABA_B_Rs (Vogt and Nicoll 1999). In a similar manner, activation of neocortical NGFC and SOM INs can silence excitatory synaptic inputs to PCs through GABA_B_Rs (Oláh et al. 2009; Urban-Ciecko et al. 2015). In the hippocampus, optogenetic approaches have revealed that SOM OLM INs selectively suppress mEC input to CA1 and enhance intrahippocampal information flow via disinhibition of the Schaffer collaterals, leading to impairment of mnemonic processing (Markwardt et al. 2011).

### Temporal control and synchronization of neuronal activity by INs

A key determinant of information transfer between neurons is synchrony, both brief synchronization during neuronal recruitment or longer-lasting network oscillations. There is mounting evidence that different IN types play a critical role in both these forms of synchronization. Inhibition has been shown to enhance the temporal precision of neuronal activation; in particular, PV BCs recruited in feedforward circuits with short latency can efficiently restrict the magnitude and duration of afferent EPSPs, thereby greatly enhancing the temporal precision of PC activation by afferent inputs (Pouille and Scanziani 2001). In a similar manner, feedback circuits efficiently limit the magnitude and duration of local excitation and thereby reduce the number of activated cells but increase their temporal synchrony. Under these conditions, highly activated PCs and recruited INs can suppress the activation of less excited neighbors through feedback and lateral inhibition, producing a “winner-takes-all” scenario (Sambandan et al. 2010).

Synchrony of neurons to network oscillations can occur over a variety of frequencies but in the hippocampus, most notably at theta (4–12 Hz), gamma (40–90 Hz) and ripple frequencies (150–200 Hz), which have been extensively reviewed (Buzsáki 2002; Buzsáki and Wang 2012). It has long been known that there is a central role of inhibitory mechanisms in the generation of network oscillations, including local feedback microcircuits, mutual inhibition and a long-range coupling. The dominance of a given IN to act as pacemaker for a particular frequency band relies heavily on their intrinsic and synaptic properties, which have been best studied in PV and SOM INs in the context of gamma and theta oscillations, respectively. The fast-spiking phenotype, rapid synaptic conductance and short feedback loop enable PV BCs to generate oscillations at high, gamma and ripple frequencies (Han et al. 1993; Kneisler and Dingledine 1995a, b). Mutual inhibition among PV BCs can further promote the coherence of oscillatory synchronization (Bartos et al. 2001; Vida et al. 2006), although it is not essential for gamma oscillation generation (Wulff et al. 2009). In contrast, the slower discharge and synaptic conductances combined with a longer feedback loop predestine OLM INs for lower frequency, theta oscillations (Chapman and Lacaille 1999; Gloveli et al. 2005; Huh et al. 2016). Finally, long-range connectivity of interneurons may synchronize oscillatory activity of neuronal populations across brain areas and contribute to the efficient transfer of information between the regions (Katona et al. 2017).

### Conclusions

In summary, we described here an updated and systematic list of all known hippocampal INs with respect to their efferent and afferent fields, synaptic connectivity and neurochemistry. Further studies will undoubtedly add to, extend and enhance the number and known properties of hippocampal INs. Indeed, the increased utilization of optogenetic and transgenic approaches will certainly facilitate our understanding of the connectivity and function of hippocampal INs. Answering the many open questions and areas of uncertainty we have highlighted will serve to better understand the function of local information processing in health and disease.
